# Human Metapneumovirus Infections during COVID-19 Pandemic, Spain

**DOI:** 10.3201/eid2904.230046

**Published:** 2023-04

**Authors:** Maria L. García-García, Elena Pérez-Arenas, Pedro Pérez-Hernandez, Iker Falces-Romero, Sara Ruiz, Francisco Pozo, Inmaculada Casas, Cristina Calvo

**Affiliations:** Severo Ochoa University Hospital Leganés, CIBERINFEC ISCIII, Madrid, Spain (M.L. García-García, S. Ruiz);; La Paz University Hospital, Madrid, Spain (E. Pérez-Arenas, P. Pérez-Hernandez);; La Paz University Hospital, IdiPaz Foundation, CIBERINFEC ISCIII, Madrid (I. Falces-Romero, C. Calvo);; Severo Ochoa University Hospital Leganés, Madrid (S. Ruiz);; National Center Microbiology, CIBERESP, Madrid (F. Pozo, I. Casas)

**Keywords:** COVID-19, SARS-CoV-2, severe acute respiratory syndrome coronavirus 2, viruses, respiratory infections, zoonoses, human metapneumovirus, outbreak, children, Spain, *Suggested citation for this article*: García-García ML, Pérez-Arenas E, Pérez-Hernandez P, Falces-Romero I, Ruiz S, Pozo F, et al. Human metapneumovirus infections during COVID-19 pandemic, Spain. Emerg Infect Dis. 2023 Apr [*date cited*]. https://doi.org/10.3201/eid2904.230046

## Abstract

We describe an unusual outbreak of respiratory infections caused by human metapneumovirus in children during the sixth wave of COVID-19 in Spain, associated with the Omicron variant. Patients in this outbreak were older than usual and showed more hypoxia and pneumonia, longer length of stay, and greater need for intensive care.

Respiratory infections caused by human metapneumovirus (hMPV) are typically epidemic during February–April. hMPV infections cause bronchiolitis and recurrent wheezing episodes and are responsible for hospitalizations mainly in children <2 years of age (*1*). The COVID-19 pandemic has changed the epidemiology of respiratory viral infections, modifying the classic seasonality of respiratory syncytial virus (RSV), influenza, and other viruses (*2*–*4*). In Spain, an outbreak of pediatric hMPV infections was identified in November–December 2021, coinciding with the sixth wave of COVID-19 in the country. This study aimed to describe this outbreak and to compare it with previous hMPV outbreaks, before the COVID-19 pandemic.

We retrospectively collected data from respiratory hMPV infections requiring hospitalization in children <18 years of age during October–December 2021 in La Paz University Hospital (Madrid, Spain) and analyzed clinical characteristics. We performed multiple PCR respiratory panels as standard clinical practice in children in whom infection by RSV, influenza, and SARS-CoV-2 had previously been ruled out by rapid test or PCR.

We obtained data from a systematic prospective study conducted over 15 years in all hospitalized children with respiratory infections in Severo Ochoa University Hospital (Madrid) using multiple PCR panels performed at the National Center for Microbiology. This study was approved by the hospital ethics committee. We compared clinical data from hospitalized patients in 2021 with our historical series of hMPV infections collected during 2005–2020, before the COVID-19 pandemic. 

We analyzed data from 48 patients with hMPV infection during October–December 2021; of those, 56.3% were male and 54% were >2 years of age, and median age was 22.7 (interquartile range [IQR] 7.1–34.9) months. Twenty-nine (60%) of the cases were detected in December and 1 in October. In 19 cases (39%), we detected co-infection with other viruses, mainly adenoviruses and human coronaviruses. A total of 34 (70.8%) case-patients had fever >38°C; in addition, 41 (85.4%) had hypoxia, and 34 (70.8%) had an infiltrate visible in their chest radiograph; radiography was not performed in 11 cases. The most frequent diagnoses were pneumonia (35%), bronchiolitis (27%), and episodes of wheezing (16%). Antimicrobial drugs were prescribed in 26 cases (54%). Seven patients (14%) required pediatric intensive care unit (PICU) admission; 2 needed mechanical ventilation. The median duration of fever was 3 (IQR 2.7–5) days, hypoxia 5 (IQR 3.5–7.5) days, and admission, 6 (IQR 4–8) days.

We compared the data from those recent patients with data from 498 cases of hMPV infection at the hospital during 2005–2020. During that period, 9 (1.8%) cases were detected in November–December and 453 (90.9%) during February–May. Patients in the 2021 season were older than those from the previous 15-year period and had significantly higher rates of hypoxia, pneumonia, antimicrobial drug treatment; they also had longer durations of fever, hypoxia, hospital admission, and PICU admission (Table).

**Table:** Comparison of the clinical characteristics of children with human metapneumovirus respiratory infections requiring hospitalization during the COVID-19 pandemic (2021) and previous seasons, Spain

Clinical data	2021, n = 48	2005–2020, n = 498	OR (95% CI)	p value
Age, mo	22 +15.9	13 +17.2	NA	0.002
Sex, no. (%)				
M	27 (56.2)	289 (58.0)	NA	0.875
F	21 (43.8)	209 (42.0)	NA	
Temperature >37.9ºC	34 (70.8)	342 (68.7)	NA	0.734
Highest temperature	**39.0** +**0.5**	**38.7 +0.6**		**0.008**
Hypoxia, oxygen saturation <93%, no. (%)	**41 (85.4)**	**331 (66.5)**	**3.9 (1.4–9.0)**	**0.002**
Chest infiltrate, no. (%)	**34 (70.8)**	**184 (36.9)**	**10.2 (3.1–32.9)**	**<0.001**
Antimicrobial treatment, no. (%)	**26 (54.2)**	**123 (24.7)**	**3.2 (1.8–5.6)**	**<0.001**
Viral co-infection, no. (%)	19 (39.6)	208 (41.8)	NA	0.632
Time of illness, Nov–Dec, no. (%)	**47 (97.9)**	**9 (1.8)**	NA	**<0.001**
Diagnosis, no. (%)				<0.001
Wheezing episode	8 (16.7)	288 (57.8)	NA	NA
Bronchiolitis	13 (27.1)	144 (28.9)	NA	NA
Pneumonia	17 (35.4)	44 (8.8)	NA	NA
Blood tests				
Leucocytes, cells/mm^3^	11,140 +5,530	12,112 +4,620	NA	0.589
C-reactive protein	40 +44	37 +54	NA	0.698
Outcome				
Duration of stay, d	**6.8 +4.8**	**4.6 +7.1**	NA	**0.004**
Duration of fever, d	**3.9 +2.4**	**2.7 +1.8**	NA	**0.001**
Duration of hypoxia, d	**6.2 +4.2**	**2.9 +2.1**	NA	**<0.001**
PICU admission, no. (%)	**7 (14.6)**	**13 (2.6)**	**5.1 (1.8–8.0)**	**0.004**

*Values are mean (SD) except as indicated. Bold text indicates statistically significant differences. NA, not applicable; OR, odds ratio; PICU, pediatric intensive care unit.

In November–December 2021, during the sixth wave of COVID-19 in Spain, the country experienced an extemporaneous hMPV outbreak at the time when RSV epidemic was usually observed. This outbreak of hMPV infections affected children older than were usually affected in previous years; we also observed a more severe clinical course and higher rates of hypoxia, pneumonia, and admission to PICU than historically. 

We considered that there may have been competition between respiratory viruses that could justify the delay in the RSV outbreak (*5*), which occurred in summer (June–July) 2021 in Spain; such competition was not the case for the hMPV outbreak, which coincided with spread of the Omicron variant of SARS-CoV-2. One possible explanation is relaxation of social distancing measures or the extreme contagiousness of Omicron. The increased severity of illness could be partly explained by the absence of hMPV infections in the previous 2 years, resulting in a susceptible population of older children who had not had previous hMPV infections and therefore had no immunity. In previous seasons, children >1 year of age were immunized by previous infections or even through residual maternal protection; this protection did not exist in 2021, and older children were infected. In conclusion, this outbreak illustrates that clinicians should be aware of potential differences in the epidemiology of other viral respiratory infections during and after the COVID-19 pandemic.
